# The English as a Foreign Language Learners’ Psychological and Emotional Perceptions on Technology Integration in Language Classrooms

**DOI:** 10.3389/fpsyg.2022.906750

**Published:** 2022-06-20

**Authors:** Xiao Yu

**Affiliations:** Foreign Language School, Xi’an University of Finance and Economics, Xi’an, China

**Keywords:** technology integration, technology literacy, technical problems, technology-based learning activities, learners’ beliefs

## Abstract

Recently, the use of new educational technologies in language teaching development is expanding rapidly. Educational technologies attach new features to the learning environments. The integration of educational technologies in language teaching has been received special attention among language researchers. In so doing, the present study investigated language learners’ perceptions of the integration of innovative educational technologies in their language classrooms. The sample comprised 301 English as a foreign language (EFL) students with different academic qualifications from Shaanxi Province (299) and other provinces (Fujian province = 1, Sichuan province = 1) in China. To gather the necessary data, the researcher conducted a technology integration questionnaire and a focus group interview. The results of obtained data demonstrated that the language learners had positive attitudes toward using technology in their classrooms. However, the findings of the interviews indicated that learners had some problems such as lack of technology literacy and inadequate assess to facilities in participating in technology-based classroom activities.

## Introduction

Today, the advent of new educational technologies has created a new type of creative, active, and interactive learning so that everyone is able to access the information they need anywhere and anytime (Bernard et al., 2014). In addition, the increasing advancement of technology has caused many changes in the social and cultural aspects of our lives. On the one hand, the world today is rapidly becoming an information society, a society in which access to knowledge and useful use of knowledge play a decisive and pivotal role. Therefore, the present era is called the information era that life is not possible without information. An era in which information is visible in all walks of human life and the criteria for the progress and development of human societies are determined by information and the extent to which it is achieved (Higashi et al., 2017). On the other hand, the most important factor and indicator of the progress of countries is their scientific and educational development. Education is not an exception and undergoes a fundamental change with the advent of information technology (Hood et al., 2015). The development of technologies is so dramatic and pervasive that their effects on education cannot be ignored. They have led to the emergence of smart schools, with the use of software and educational technology capabilities (Hsu et al., 2008).

Over time, conventional approaches to second language teaching have been challenged by technological innovations. The general findings of research studies on the integration of educational technologies indicate that technology might provide opportunities to create an adequate and powerful learning environment; an environment that might increase language learners’ motivation, willingness to communicate, and independence (Alraimi et al., 2015; Huang and Chou, 2015; Lee and Lai, 2017). For instance, Abeysekera and Dawson (2015) noted that technology could facilitate fundamental change in teaching and learning. They also argued that using group-learning technology improves curriculum integration and teacher communication.

While technology has many advantages, some barriers stop language teachers from accessing educational technologies in language classrooms. One of the key and effective variables in the integration of educational technologies is the technological beliefs of language learners (Mellati and Khademi, 2015). Language learners are considered as one of the most central factors for the development of academic achievement (Bielak and Mystkowska-Wiertelak, 2020; Derakhshan, 2021; Liu and Song, 2021). Because of the several emotional roles of language learners in the classroom, their psychological and emotional perceptions can influence the emotional status of the class and affect learners’ educational experience (Arnold and Fonseca Mora, 2011; Reschly and Christenson, 2012; Mellati et al., 2015). Technology integration is an element that affects language learners’ psychological and emotional perceptions (Dewaele et al., 2019). Based on the previous studies, instruction is associated with dissatisfaction, anxiety, and instruction inefficacy (Dewaele and Pavelescu, 2021). Therefore, technology integration might lead to harmful outcomes for educators as well as the instruction quality. Potential outcomes of educator emotional status can decrease the level of satisfaction, the degree of dedication, and higher education (Reschly and Christenson, 2012; Fathi and Derakhshan, 2019). Emotional perception means the mood wherein people experience bodily and psychological fatigue after high stress working which is considered as a symptom of emotional fatigue (Ghanizadeh and Royaei, 2015; Gabryś-Barker, 2021). Emotional status should be usually checked in careers presenting human services and education as one of those careers (Ghanizadeh and Royaei, 2015). In comparison to different careers, in education emotional status is mostly experienced and therefore leads to tension, and speeds up educator’s anxiety (Greenier et al., 2021). Language learners’ emotional status is described as emotions of no power in an attempt to train learners and build a desirable atmosphere in school for them. It means no passion to ready the lessons. It means that they have trouble encouraging themselves to do a task (Gregerson et al., 2014). As a results, educators require holding positive emotions and great degrees of motivation pertaining to their classroom activities to achieve their educational targets (Ghanizadeh and Royaei, 2015; Jin et al., 2021) and teaching is a crucial component of nurturing a prosperous generation. However, studies display an agitating excessive number of language teachers struggling with technology integration difficulties worldwide (Lake, 2013; Li, 2020).

Generally, researchers have reported that technology is more likely to be used by language learners who have a positive attitude toward learning technology (Miyazoe and Anderson, 2010; Truong, 2016). The modern concept of information technology and its impact on different facets of life has led to the appearance of some basic modifications in the relationship of human societies (Barak et al., 2016). This phenomenon has rapidly affected human desires and created new needs. Today, the importance of education system that is customized to the requirements of the individual and society is most felt (Chao and Lo, 2011; Alraimi et al., 2015). New technologies can be compared to rain, which if they rain in the right place can cause nature to grow and revive, but if they rain in the wrong place can cause floods (Mellati and Khademi, 2014b). The expansion of educational technologies in an efficient educational system is not only a choice but also an undeniable necessity and is an important step in the reformation of educational systems. However, Barak et al. (2016) point out that despite language learners’ positive attitudes, they rarely integrate educational technologies in their classrooms. They claim that insufficient technical support and teachers’ beliefs are major obstacles to the language learners’ integration of educational technologies in education (Aysel, 2014).

Considering the above-mentioned points, researchers and teachers should apply different educational technologies in such a way that human interactions are not disturbed and education is not restricted to the mere use of some mechanical tools and equipment (Alraimi et al., 2015; Bozkurt and Keefer, 2017; Mellati and Khademi, 2018). In line with this policy, identifying the characteristics of efficient and effective education is of particular importance (Bernard et al., 2004; Alavi et al., 2021). If the culture of teaching and learning does not change in the educational system, not only will the introduction of information technology into this system not cause any change, but it will strengthen the conservative traditions of education because it is not information technology alone that will change. Human beings are considered as the main factor for change (Bueno-Alastuey and Lopez Perez, 2014; Hockly, 2015).

There is an agreement that the development in educational technologies have motivated the creation of new teaching strategies (Teo, 2011). Therefore, the Chinese government has invested profoundly in creating required infrastructure within educational contexts and providing training opportunities for EFL teachers. For example, Education information: 10 years development plan (2011–2020) was one of the Chinese plan that was issued in 2012. This plan explicitly put forward the requirement of integrating technology into teaching and learning across subjects, including facility investment, technical supports, and teachers’ technological and pedagogical skills to ensure technology integration into education (Li and Ni, 2011). Chinese policy makers argued that the use of ICT as a teaching and learning tool has been acclaimed as a catalyst for educational transformation by and teachers (Yang, 2012), more so in EFL contexts. Integration of ICT into EFL teaching and learning greatly facilitates the creation of an authentic language-rich environment, bridges the gap derived from the identities of teachers and non-native speakers (Wang and Coleman, 2009), and promotes inter active language teaching and learning activities (Golonka et al., 2014).

However, there have been many debates about the integration of educational technologies in education. Generally, technology has been considered as a technical tool to raise the level of education (Watson et al., 2016; Zhou, 2016). This puts a clear and significant point in front of us; there is an urgent need for the development and professionalization of teachers and students who want to use technology in their classrooms (Bernard et al., 2004; Trust et al., 2017). Therefore, it is required to pay special consideration to the fact that before providing the possibility of mixing these two categories, a suitable cultural-scientific background should be created for it. One of these factors that need special attention is learners’ beliefs about employing technology in educational settings.

## Review of Literature

### Effects of Technology on Language Learning

In recent years, an English teacher was the only one to provide credible English content, and the text was the only source in English language teaching environments, but with advances in computer and the Internet technologies, traditional approaches to language teaching and learning have been challenged or replaced with new and innovative approaches (Bernard et al., 2014; Hew and Cheung, 2014; Kent et al., 2016; Burke and Fedorek, 2017; Mellati et al., 2018). Using technology in English language classes, language teachers can convey the content more easily and effectively to the language learners (Phan et al., 2016). They stated that the use of technology has great potential for changing language-teaching methods. Huang and Chou (2015) stressed that by using technology language learners might control their own learning processes and have access to a huge amount of information that is beyond the control of teachers. Hsu and Wang (2014) examined the characteristics of multimedia computers in foreign language teaching and showed that the use of multimedia makes the class more attractive. They also stated that teaching English with the help of multimedia could increase language learners’ motivation, optimize the classroom environment, improve listening and speaking skills, promote ideas in the target language, and stimulate their desire to communicate (Hsu et al., 2008).

Technology also helps the social aspect of language to communicate with real speakers, which usually happens outside the classroom (Viberg and Grönlund, 2015; Littlejohn et al., 2016; Ismaili and Ibrahimi, 2017; Mellati et al., 2021). Using technology, language learners’ opportunities and their chances of taking responsibility for their learning might increase (Kent et al., 2016). Given this background, it can be concluded that technology can help language learning and, if used properly, make classes more attractive to language learners. Language learners’ beliefs can be influenced by their teachers’ beliefs about technology (Kent et al., 2016; Watson et al., 2016; Chen Hsieh et al., 2017).

### Language Teachers’ Beliefs

Numerous studies have highlighted the important function of technology in the development of language skills, independence, and motivation of language learners (Stewart et al., 2011; Henrie et al., 2015; Hood et al., 2015; Tsai et al., 2016; Zhang, 2016; Chen Hsieh et al., 2017; Mellati and Khademi, 2019). However, it has been proposed that learners’ acceptance and rejection of technology is influenced by their cognition and belief. It suggests that the perceived ease of use and perceived usefulness are two core factors in explaining user attitudes toward using, behavioral intentions, and actual use. The integration of educational technologies not only helps teachers and learners in the classroom, but also offers opportunities for language learners outside the classroom (Ginns and Ellis, 2009; García-Sánchez, 2016). A few studies have reported that language teachers do not believe in technology integration and have not integrated technology to help language teaching and curriculum development in the classrooms. These perspectives might influence language learners’ perceptions of technology integration. They also claimed that their use of technology has often been superficial and limited (Greene et al., 2015; Henrie et al., 2015; Ko, 2017).

For instance, Liu et al. (2017) proved that pre-service English teachers who had undergone training courses about the integration of educational technologies were not fully prepared to implement technology in their classrooms, and it appears that there is a gap between what pre-service teachers have learned in their technology training course and actual use. One potential factor associated with teachers’ limited use of technology is their educational beliefs. They suggested that teachers’ beliefs are the most important factor in how they use technology (Littlejohn et al., 2016). Based on the results of previous research, teachers choose technologies that are consistent with their curriculum variables and teaching methods (teaching strategies) and their beliefs about “good” teaching (Mellati and Khademi, 2014a; Lo and Hew, 2017; Trust et al., 2017; Wenming and Zhang, 2017).

Technology tools such as computers, tablets, or interactive whiteboards do not impose a particular educational approach on teachers; rather, each of these tools allows them to implement a wide range of teaching and learning approaches (Greene et al., 2015; Tsai et al., 2016). In other words, the role of technology in teachers’ classrooms is related to learners’ beliefs and perceptions of technology and their functions in language teaching and learning processes (Higashi et al., 2017; Liu et al., 2017). They also stated that to succeed in applying educational technologies in language classrooms, language teachers’ negative beliefs should be identified and corrected, and their positive beliefs must be strengthened (Stewart et al., 2011; Phan et al., 2016). Many studies show teachers and learners have positive beliefs about technology-based language learning in the classroom (Snodin, 2013; Vu et al., 2015). They concluded that most English teachers have a positive perception of technology use and consider computer-assisted language learning to be effective in increasing motivation, independence, self-confidence, and the ability to learn different cultures (Abeysekera and Dawson, 2015; Bozkurt and Keefer, 2017; Trust et al., 2017).

Since computer language learning facilitates access to information, professional development, the use of different educational approaches, and English language assessment, teachers also believed that technology was an important, facilitative, and interactive tool in English language teaching (Vu et al., 2015; Burke and Fedorek, 2017; Higashi et al., 2017). However, not all EFL teachers and learners have positive attitudes toward the integration of educational technologies in language classes. Some of them did not approve of the use of educational technologies in language classrooms (Yang et al., 2017). These teachers and learners feel more secure and confident when they work in a conventional learning environment (without the use of technology) (Viberg and Grönlund, 2015). This reluctance or even resistance to the integration of educational technologies in the classroom may be due to skepticism and low self-esteem (Yang et al., 2017). No matter what was the source of their beliefs, they transfer this reluctance to their language learners (Hockly, 2015). However, studies that report negative beliefs are relatively few compared to studies that show teachers’ positive attitudes toward the integration of educational technologies in language classrooms (Chao and Lo, 2011). Therefore, the first step to determine language learners’ beliefs about integrating technology into language classrooms is identifying their teachers’ beliefs. Teachers’ beliefs about educational technologies might affect learners’ learning styles and preferences (Hsu and Wang, 2014).

### Learning Styles

Style refers to a person’s preferred way of using his abilities and thus, differs from his ability. Style is a very important factor in recognizing individual differences in language performance and their preferences when they think, learn, teach, or do different things (Hsu et al., 2008). From Ko’s (2017) point of view, learning styles reflect those fixed characteristics and behaviors that appear in the way our classrooms are managed. Style is what describes us and guides our learning processes and affects language learners’ ability. He also defined learning styles as reflecting the integration of language learners’ theoretical assumptions and practical activities. Studies of learning styles have shown that if they are trained according to their preferences in receiving and processing information, their academic achievement will increase (Yang, 2014). Research results show that adapting educational materials to meet the different learning needs of students is beneficial for them (Ismaili and Ibrahimi, 2017). This requires us to know their learning and cognitive styles and to know what kind of content is needed for each style (Yanguas, 2010; Liu et al., 2017).

Efforts to improve and enhance education through information and communication technology require a clear understanding of the role of language learners in education (Zimmerman, 2008). Many factors are effective in the integration of educational technologies by language learners in language teaching processes and one of these factors is language learners’ beliefs (Truong, 2016). A review of the research background in teacher education shows that teachers’ teaching methods and their beliefs about the integration of educational technologies have already been studied separately in different studies. However, studies have rarely been conducted to investigate the language learners’ beliefs about technology integration in language classrooms. Undoubtedly, the lack of research in this area is a logical reason to examine the language learners’ beliefs about technology.

### Research Questions

To solve the abovementioned problem, the present study was conducted to investigate Chinese language learners’ beliefs about the integration of educational technologies in language teaching contexts and answer the following questions:

**RQ1**: What are Chinese English learners’ perceptions about integrating technology in language classrooms?

**RQ2**: What are the possible advantages and barriers of technology integration in Chinese English language classrooms?

## Method

### Participants

The sample comprised of 301 English students with different academic qualifications including both genders (male = 95/31.5%, female = 68.5%) with their ages ranging from 17 to 22, including 25 junior college language learners, 263 undergraduates, and 13 postgraduates, studying in different majors of English language such as English Translation, English Literature, and English Language Teaching. To generalize the research results, respondents of this research were recruited from Shaanxi Province (299) and other provinces (Fujian province = 1, Sichuan province = 1) in China. The required data were collected through Wenjuanxing (a software used to make questionnaires) via the Wechat phone app. Consent was given to all participants. The collected data was also based on participants’ willingness.

### Instruments

#### Technology Integration Questionnaire

The first instrument that the researcher used in the current research study was Technology Integration Questionnaire. The questionnaire was adopted from Attributes of Diffusion of Innovation Questionnaire developed by Rogers (1995) and Stages of Concern Questionnaire developed by Hall (1987). The Technology Integration Questionnaire was employed to identify the patterns of language learners’ present worries about an innovation that refers to the integration of instructional technologies in language teaching classrooms. The items of this 27-item questionnaire were written in the form of statements about personal feelings or attitudes in a three Likert-scale format that starts from *Agree (A)* and ends to *Disagree (D)*. It focuses on seven factors: learners’ motivation to use technology, the effectiveness of used technology, learners’ engagement in technology-based settings, language learners’ learning autonomy, language learners’ technology literacy, learning style, their technophobia. The participants showed their attitudes toward each item and the concept under question as well. To verify the reliability index of the preliminary form of the questionnaire, the researcher piloted its first edition with 50 language learners of a similar context. The researcher used the Cronbach Alpha coefficient, the results of this analysis showed the reliability index of 0.79 (*r* = 0.79).

The categories and their related questions are presented in Table 1. Five questions determined language learners’ perceptions of the effectiveness technology integration; four questions determined language learners’ perceptions of engagement in technology-supported educational environments; eight questions determined language learners’ perceptions of motivation in technology-supported learning environments; three questions checked their learning autonomy; one question determined their perceptions of the compatibility of technology-based learning environments to language learners’ learning styles; two questions checked language learners’ technophobia; and four questions checked their technology literacy.

**TABLE 1:** The categories of the questionnaire and the questions under every category.

Category	Questions	Type of question
Effectiveness	2. Integrating technology improves my classroom performance in English language.	Likert scale
	4. Integrating technology increases my learning productivity English-language related.	Likert scale
	11. I believe that technology is easy to integrate to my learning schedule in English-language related.	Likert scale
	13. I have had a great deal of opportunity to try various technology applications.	Likert scale
	17. I would have no difficulty telling others about the results of integrating technology in my English-language related classroom activities.	Likert scale
Engagement	19. I have seen what others do when integrating technology in their learning.	Likert scale
	20. It is easy for me to observe others using technology in my university.	Likert scale
	21. I would like to help other students in their use of the technology integration.	Likert scale
	23. I would like to develop working relationships with my peers both inside and outside my university using technology in our classroom activities.	Likert scale
Motivation	5. Integrating technology is completely compatible with all aspects of my classroom activities. +	Likert scale
	6. Integrating technology is completely compatible with my current situation. +	Likert scale
	7. I think that integrating technology fits well with the way I like to learn English. +	Likert scale
	9. I believe that technology is difficult to integrate to my learning schedule.	Likert scale
	10. Integrating technology is often frustrating to me.-	Likert scale
	14. I know where I can go to satisfactorily try out various uses of technology.	Likert scale
	18. The results of integrating technology in my classroom activities are apparent to me.	Likert scale
	27. I would like to know how technology is better than what we have now.	Likert scale
Learning autonomy	1. Integrating technology makes it easier for me to do my English- language related classroom activities.	Likert scale
	3. Integrating technology gives me greater control over my English- language related classroom activities.	Likert scale
	12. Learning to integrate technology in my learning activities is easy for me.	Likert scale
Learning style	8. Integrating technology fits into my learning style in my English classes.	Likert scale
Technophobia	15. Before deciding whether to integrate technology applications, 1 was able to properly try them out.	Likert scale
	16. I was allowed to integrate technology on a trial basis long enough to see what it can do for learning English.	Likert scale
Technology literacy	22. I have a very limited knowledge of the technology integration.	Likert scale
	24. I am concerned about my inability to manage all that the technology requires.	Likert scale
	25. I am concerned about time spent working with non-academic problems related to the technology integration.	Likert scale
	26. Coordination of tasks and people in a technology-based classroom is taking too much of my time.	Likert scale

#### Focus Group Interview

To understand the language learners’ perceptions about the integration of technology into language classrooms, the researcher conducted an interview with 15 participants. The main questions of this focus group interview were related to the participants’ motivation, attitudes, and experience of technology-based learning environments. In this interview, which lasted 30 min, the researcher attempted to determine the language learners’ attitudes over the integration of educational technologies in language classrooms.

Some questions of the interview were as follow:

• Do you consider technology integration in English-language-related classroom as an advantage or disadvantage? Why?
• What are the difficulties of employing educational technologies in your opinion in English-language-related issues?

### Data Collection Procedures

To investigate the language learners’ attitudes toward technology integration in language classrooms, the researcher conducted a questionnaire survey. By distributing the valid questionnaire online through Wenjuanxing (software to make a questionnaire and select data) via Wechat, the researchers collected the required data in the middle of November 2021. Altogether, 301 valid questionnaires were gleaned from different colleges, institutes, and universities in China. To meet the purposes of the current study, language learners were notified of how to fill out the questionnaires and report their responses and assured that their responses were only used to meet research objectives and would remain confidential. They were also informed of their rights to leave the study freely if they felt any discomfort. The questionnaire asked language learners to present truthful responses about their technology-based learning experiences. The researchers clearly stated in the questionnaire that the questions do not have definite answers, and language learners only needed to choose what they think is right, and that the answers would in no way have any effect on their future. As the participants made no contact with the researcher, there were no interest conflicts between the researcher and respondents. Then, the collected responses were double-checked before being sent to SPSS software for further statistical analysis. At the final step, the probe into the research questions was conducted.

In the next phase, the researchers conducted a 30-min focus group interview with 15 language learners. It was started by asking questions about implementing technology in their classrooms. They were also open to talk about their personal feelings and attitudes about technology. To conduct content analyses, the researcher transcribed the interviewees’ answers. The obtained qualitative and quantitative data were considered for further analyses. To find the answer to the first research question, the researcher employed descriptive analysis. To discover the answer to the second question, the researcher employed content analysis. The results of the focus group interview were the qualitative data of the study.

## Results

### Quantitative Data Analysis

The researcher used descriptive analysis to find the answer to research question number one. In the following tables, the results of these analyses are presented. To smooth the progress of explanation of the obtained results, the researchers merged the values in *Strongly Agree (SA)* and *Agree (A)* in the results into *Agree* and merged the values of *Strongly Disagree (SD)* and *Disagree (D)* into *Disagree*.

#### Effectiveness of Technology Integrated Learning Environment

This section was designed to determine language learners’ attitudes toward the effectiveness of different technologies in their language classrooms (see Table 2).

**TABLE 2:** Frequency and percent of the questions related to the efficacy of technology integration.

Questions	Frequency	Percent	Frequency	Percent	Frequency	Percent
2	247	82	45	15	9	3
4	229	76.1	60	19.9	12	4
11	203	67.5	82	27.2	16	5.3
13	210	69.8	75	24.9	16	5.3
17	158	52.5	101	33.6	42	14

The results of Table 2 show that 82 percent of language learners their language performance has been improved after participating in a technology-based learning environments (question 2). More than 60 percent of the learners believed that technology integration has increased their leaning opportunities in the classrooms (question 13).

The results of Table 3 show that approximately all of the participants accepted the efficacy of employing technology in language classrooms.

**TABLE 3:** Descriptive statistics of the questions related the efficacy of technology-integrated classrooms.

Questions	N	Minimum	Maximum	Mean	Std. deviation
2	301	1	5	1.87	0.794
4	301	1	5	2.01	0.808
11	301	1	5	2.19	0.813
13	301	1	5	2.17	0.798
17	301	1	5	2.48	0.919
Valid N (listwise)	301				

#### Learners’ Engagement in Technology Integrated Learning Environment

The next category is language learners’ attitudes toward engagement in technology-supported language learning contexts.

Table 4 indicates that the learners’ responses to the questions of 19, 20, 21, and 23 show that language learners agreed that integrating technology into their language classrooms increase their engagements.

**TABLE 4:** Frequency and percent of the questions related the learners’ engagement in of technology integration learning environments.

Questions	Agree		Uncertain		Disagree	
	Frequency	Percent	Frequency	Percent	Frequency	Percent
19	200	66.5	79	26.2	22	7.3
20	162	53.8	98	32.6	41	13.7
21	192	63.8	93	30.9	16	5.4
23	227	75.4	65	21.6	9	3

The mean value for question number 23 in Table 5 shows that more than 75 percent of the participants agreed that integrating technology increases their opportunities in and outside of the classroom to engage in language activities.

**TABLE 5:** Descriptive statistics of the questions related the engagement of technology-integrated classrooms.

Questions	N	Minimum	Maximum	Mean	Std. deviation
19	301	1	5	2.23	0.865
20	301	1	5	2.46	0.918
21	301	1	5	2.23	0.838
23	301	1	5	2.07	0.745
Valid N (listwise)	301				

#### Learners’ Motivation in Technology Integrated Learning Environment

Eight questions of the questionnaire were designed to elicit language learners’ perceptions of motivation to participate in technology-based language course and related classroom activities. The results of Table 6 demonstrate that language learner were motivated to participate in technology-based learning contexts. In addition, the answers to questions 9 and 10 that were counterbalance questions show the learners’ attention in filling the questions and validity of the answers. More than 85 percent of the learners stated that they had no difficulty integrating technology to their learning schedules. Their answers to question 10 show that more than 80 percent of the learners did not frustrate using educational technologies in their language classrooms.

**TABLE 6:** Frequency and percent of the questions related to the learners’ motivation in technology integration learning environments.

Questions	Agree		Uncertain		Disagree	
	Frequency	Percent	Frequency	Percent	Frequency	Percent
5	166	55.1	101	33.6	34	11.3
6	179	59.5	92	30.6	30	10
7	213	70.7	75	24.9	13	4.3
9	97	32.2	86	28.6	118	39.2
10	106	34.6	80	26.6	115	31
14	194	64.4	91	30.2	16	5.3
18	187	62.1	98	32.6	16	5.3
27	239	79.4	59	19.6	3	1

The results of Table 7 reveal that language learners’ motivation to technology-integrated classrooms is near intermediate value. However, the overall motivation of the participants is positive. Teachers talk about motivation in many ways. Some talk about motivation in a particular area, such as a greater desire to write or work on competitive skills. Others have spoken of more general motivational effects.

**TABLE 7:** Descriptive statistics of the questions related to the learners’ motivation in technology-integrated classrooms.

Questions	N	Minimum	Maximum	Mean	Std. deviation
5	301	1	5	2.38	0.954
6	301	1	4	2.33	0.877
7	301	1	5	2.14	0.788
9	301	1	5	2.98	1.094
10	301	1	5	2.99	1.152
14	301	1	5	2.24	0.809
18	301	1	5	2.28	0.794
27	301	1	5	1.92	0.755
Valid N (listwise)	301				

#### Learning Autonomy

The rapid feedback provided by the computer to the language learners’ satisfaction the feeling of accomplishing a task and the power gained in the course of technology have a significant effect on language learners’ independence. These questions had been designed to elicit language learners’ perceptions of autonomy in technology-integrated classrooms. The classroom as a group or social system has all the features of social systems that are considered by behavioral scientists. Classroom management is the responsibility of the teacher, who must manage the classroom to achieve the goals before training or any educational or behavioral action. Today, one of the most important challenges for a teacher in the classroom is effective and correct communication with learners. On the other hand, meaningful communication is the key to the effective use of many management practices in the classroom.

The results of Table 8 show that near 80 percent of the participants agree that technology integration increases their control over the content and pace of learning. As control of the language learners increase, the course move from a teacher-centered course to a learner-centered course.

**TABLE 8:** Frequency and percent of the questions related to the learners’ autonomy in technology integration learning environments.

Questions	Agree		Uncertain		Disagree	
	Frequency	Percent	Frequency	Percent	Frequency	Percent
1	265	81.8	31	10.3	5	1.7
3	238	79	54	17.9	9	3
12	181	64.1	100	33.2	20	6.7

The results of Table 9 show the mean values of questions one and three show upper intermediate values. They support collaborative and interactive learning activities in a learner-centered learning environment.

**TABLE 9:** Descriptive statistics of the questions related to the learners’ autonomy in technology-integrated classrooms.

Questions	N	Minimum	Maximum	Mean	Std. deviation
1	301	1	4	1.75	0.704
3	301	1	4	1.94	0.774
12	301	1	5	2.29	0.859
Valid N (listwise)	301				

#### Learning Style

One of the important facts of existence is the existence of diversity among the phenomena of the universe. Humans are subject to the same rule. Language learners differ in terms of mental abilities, learning methods, style, and speed of learning, readiness, interest, and motivation to acquire knowledge and perform academic activities. Various factors cause differences between learners. Although they are different in terms of their learning styles, 67.1 percent of the participants agreed that technology could fit into their learning styles (Table 10).

**TABLE 10:** Frequency and percent of the questions related to the learners’ learning styles in technology integration learning environments.

Question	Agree		Uncertain		Disagree	
	Frequency	Percent	Frequency	Percent	Frequency	Percent
8	202	67.1	81	26.9	18	5.9

The results of Table 11 reveal that the mean value for learners’ perceptions of how much technology can cover different learning styles is intermediate.

**TABLE 11:** Descriptive statistics of the questions related to the learners’ learning styles in technology-integrated classrooms.

	N	Minimum	Maximum	Mean	Std. deviation
Learning style	301	1	5	2.21	0.816
Valid N (listwise)	301				

#### Technophobia

Two questions had been designed to check language learners’ technophobia in this study. Fear of technology and computers is an important issue in many societies, because there are many people who have a negative feeling about computers and that computers are increasingly embedded in all aspects of life, avoiding its use.

The results of Table 12 disclose that while many language learners had positive attitudes toward employing technology in language classrooms, more than 50 percent of them (69.1 in question 15 and 55.8 percent in question 16) had concern about how to use it properly.

**TABLE 12:** Frequency and percent of the questions related to the learners’ technophobia in technology integration learning environments.

Questions	Agree		Uncertain		Disagree	
	Frequency	Percent	Frequency	Percent	Frequency	Percent
15	208	69.1	81	26.9	12	4
16	168	55.8	91	30.2	42	13.9

The results of Table 13 indicate that the mean value for technophobia is in the intermediate level. When language learners’ learning styles are aligned with the employed technologies in teaching, learners’ motivation, performance, and progress increase.

**TABLE 13:** Descriptive statistics of the questions related to the learners’ technophobia in technology-integrated classrooms.

Questions	N	Minimum	Maximum	Mean	Std. deviation
15	301	1	5	2.16	0.781
16	301	1	5	2.44	0.949
Valid N (listwise)	301				

#### Technology Literacy

Technology literacy has been defined by the International Technology Education Association (ITEA) as human innovation in practice as well as the ability to manage, use, understand, and evaluate technology. In fact, technology literacy is a new type of literacy that has led to the creation, dissemination, and consumption of technology, and includes methods by which people can easily consume a variety of technologies. Technological literacy can affect how we search the world and affect individual and social life and culture, as well as lead to an optimistic outlook on the future.

Four questions had been designed to elicit language learners’ perceptions of technology literacy. The results of Table 14 show that about half of the participants agreed that they have limited knowledge in technology integration. This limited knowledge causes more than 50 percent of the participants in question 24 agreed that they are concerned about their incapability of controlling all that the technologies need.

**TABLE 14:** Frequency and percent of the questions related to the learners’ technology literacy in technology integration learning environments.

Questions	Agree		Uncertain		Disagree	
	Frequency	Percent	Frequency	Percent	Frequency	Percent
22	139	46.2	95	31.6	67	22.3
24	160	53.2	89	29.6	52	17.3
25	195	64.8	83	27.6	23	7.7
26	126	41.5	95	31.6	80	26.6

Table 15 demonstrates that the mean values for technology literacy are in intermediate level. Half of the participants agreed that technology literacy is a major concern in technology-based learning environments.

**TABLE 15:** Descriptive statistics of the questions related to the learners’ technology literacy in technology-integrated classrooms.

Questions	N	Minimum	Maximum	Mean	Std. deviation
22	301	1	5	2.63	1.033
24	301	1	5	2.51	0.992
25	301	1	5	2.23	0.875
26	301	1	5	2.73	1.047
Valid N (listwise)	301				

### The Analysis of Qualitative Data

The researcher gathered the qualitative data of the study to find the answer for question number two through an interview with 15 language learners. The researcher used the following procedure for analyzing the obtained data: the researcher read the collected data several times to discover the most important ideas. Then the researcher coded and analyzed the findings manually and subjectively; the collected data in the interview were converted into text. The researcher used open coding to analyze the transcribed transcript. Some of the central attitudes toward about the integration of educational technologies into language classrooms in the interview were as follow:

#### Encouraging Ideas About Employing Technology in Language Classrooms

Approximately all of the participants stated that integration technology into their classrooms had some advantages.

##### The Quality of the Teaching

They stated that the integration of technology improves the quality of English classes and helps them to learn English easily. *For many students, the integration of technology can help them learn English more easily, which is good for both teachers and students, and makes the classroom more efficient* (Learner #1 stated in the interview, Learners # 9 and 11 stated the same concept but in different words).

They also stated that the integration of technology is conducive to make their learning more convenient. *Technology integration can enhance the interest of English class, making learning more convenient* (Learner #13 stated in the interview, Learners # 2 and 8 stated the same concept but in different words).

Improvement in the learning progress was another issue that was highlighted in the interviews.

*In my opinion, English learning is a multi-faceted learning of listening, hearing, speaking, reading and writing, and ordinary paper textbooks cannot fully meet the learning needs. Technology integration can better enable teachers to provide students with rich resources in the teaching process, provide language environment for students through network technology, deepen students’ understanding of foreign culture, and this teaching method can also improve students’ interest in learning* (Learner #15 stated in the interview, Learner # 3 stated the same concept but in different words).

Effective use of social networking and media technologies provide extraordinary opportunities for course designers and instructors to interject emotions in the online learning environments, thus providing learning opportunities for learners to make emotional connections with classmates just as they do in the real time out of the classrooms (Burke and Fedorek, 2017).

##### Language Learners’ Motivation

The results of interview pointed out that the integration of technology encourages language learners to participate in classroom activities eagerly. *It can change the traditional teaching mode and stimulate students’ enthusiasm for learning* (Learner # 4 stated in the interview). The integration of educational technologies tools in teaching offers chances for learners to engage in real world learning, and in general, makes learning a foreign language more attractive and increases their motivation to participate in classroom exercises. Furthermore, the social media tools create a constructivist learning environment which allows learners to construct interpretations of their data and utilize their individual life experience while working as a part of a collaborative team (Alraimi et al., 2015).

##### Language Learners’ Engagement

Healthy and effective communication is the basis for the movement and promotion of language learners. Teachers in the classroom face important challenges, including motivating all students, doing group work, monitoring student behavior, and monitoring learners’ progress. Proper classroom management can enhance the teacher’s ability. Proper classroom management based on technology leads to the realization of order and strengthens internal motivation to perform classroom activities. *Technology integration can improve classroom efficiency and classroom interaction rate* (Learner # 6 stated in the interview).

Learners can use social networking to create their own learning and social communities and their new identities (Higashi et al., 2017). These online, social, and self-directed learning settings provide resources that enhance learners’ engagement in the course. There are many social media tools that can be integrated into the curriculum to support learning and provide innovative and effective directions for content delivery in both synchronous and asynchronous language learning environments (Liu and Song, 2021).

##### Vivid and Clear Learning Process

Language learners stated in the interview that employing technology helps them to understand the learning process clearly. *Modern technology can make the learning process more vivid and clear in foreign language classes* (Learner # 12 stated in the interview, Learner # 7 and 14 stated the same concept but in different words).

The digitalization of educational resources and learning materials has enabled the re-use of these resources across countries and scholarly domains. These systems focus on online social networks to create connection and to improve engagement. Social networks can create and sustain the social dimension of learning, and enhance knowledge production rather than simply providing a platform for knowledge consumption. An online course has unlimited participation and open access via the web. They provide interactive user forums to support community interactions among students and teachers (Dewaele et al., 2019).

#### Challenging Ideas About Employing Technology in Language Classrooms

##### Technology Literacy

*In my opinion, the barrier to technology integration is the computer literacy of teachers and students* (Learner # 12 stated in the interview). Most of the learners argued that the main challenge in a technology-integrated learning environment is teachers and learners’ technology literacy. They believed that their technology literacy could influence all aspects of the language teaching process from designing materials to the emotional status of language learners.

##### Management and Facilities

The learners believed that the next barrier in the integration of technology is technical support and teachers’ management.

*I think the main obstacles are management and facilities. Only when the infrastructure of technology integration becomes stable after continuous improvement and upgrading can it facilitate the follow-up work* (Learner # 12 stated in the interview, many learners stated the same concept but in different words).

Many researchers focused on this dimension and emphasized the challenges that they had in their studies, for instance, Truong (2016) argued that the low availability of technology which embraces the subcategories shortage of appropriate infrastructure and software can be the main barrier in many contexts.

Related to this aspect, a lack of common data references, definitions, and channels which impede a data and information exchange via technical means (Li, 2020), concerns about security and privacy (Hsu and Wang, 2014), restricted the access to online resources and platforms in technical manner are some other barriers that learners are faced with in their educational contexts.

Bielak and Mystkowska-Wiertelak (2020) stated that in the social dimension, the first aspect of challenges is the value of these systems on the national level. There are many differences in ethnic, national beliefs, and common understanding toward the features of these new settings. Littlejohn et al. (2016) believed that the main barrier in the social dimension relates to individual concerns. A subcategory in this respect is socialization.

## Discussion

The findings of this study suggest that language learners have positive attitudes toward employing technology in language classrooms. However, they argued that employing technology in language classroom has some advantages and some disadvantages. The results of data analysis demonstrated that teachers are the most influential curriculum implementers who can implement innovations competently or with low quality in the classroom. They play a key role in shaping learners’ beliefs about integrating technology into language classrooms. The finding is confirmed with Bueno-Alastuey and Lopez Perez (2014) who found that language teachers could encourage or discourage language learners to take part in technology-based learning activities. With the help of technology, language learners can save time in English classrooms and learn the language easily and efficiently (Higashi et al., 2017). The use of technology creates a learner-centered learning environment. Focus on language learners leads to positive changes. Researchers believed that the integration of computer technology could make classrooms an active place where real learning takes place and learners take responsibility for their learning (Hockly, 2015).

The findings of this study showed that language learners find the use of computer technology in educational courses because it not only facilitates the process of language learning and teaching, but also increases their motivation. These findings are in line with Stewart et al. (2011) which show that language learners have a positive attitude toward employing computers in teaching English. Similarly, Lee and Lai (2017) identified several benefits of using computers in English language teaching, such as improving the quality of instruction, improving assessment in language tests, and improving participation.

In contrast to Liu et al. (2017) who argued that language learners are convinced that computer-assisted language learning is useful, the results of the present study found that there were several obstacles to doing so in English language courses. The results of the interview demonstrated that technology literacy and technical problems are among the most important barriers for language learners to engage in technology-based activities in their classrooms. In consistent with the findings of the current study, Burke and Fedorek (2017) argued that technology literacy affect teachers and learners’ beliefs about employing technology in their teaching and learning contexts. These factors can be divided into internal and external categories. Internal factors include aspects of language learners’ characteristics, such as their attitudes toward the technology and their skills and knowledge. External factors include the effects of context-based factors such as technical support, computer facilities, and their teachers’ knowledge of the technology.

## Conclusion

The current study investigated Chinese language learners’ beliefs about the integration of educational technologies in English teaching contexts. The findings demonstrated that Chinese language learners had positive attitudes toward employing technologies in their classrooms. They stated that technology-integrated learning environments facilitate their learning, motivate them to participate in classroom activities, and enhance their engagement in language classrooms. However, some internal and external barriers reduce the popularity of the technology among language learners. Technology literacy, access, and technical problems are among the most important ones.

## Implications

It is not a secret to experts that the dynamism of various educational methods and their reliance on scientific bases has a major impact on the growth of learning and improving the level of learning of graduates. To reach this goal, experts in the field should do their best in designing and applying technologies through effective methods and procedures. The findings of this study should be used to increase the awareness of English teachers as foreign language and policymakers in institutions to realize that many elements, including teachers’ teaching methods and their beliefs and their students’ beliefs about technology, provide the ground for teachers. Since teachers’ beliefs influence learners’ beliefs, the findings of this study suggest that principals:

• Use technology to develop the quality of teachers’ teaching for easy and better learning of language learners.
• Use specialized teachers in technology posts such as audio-visual directors.
• Attracting teachers with higher degrees in specialized fields and familiarity with current technologies in teaching.
• Informing teachers and professors about the latest findings related to new technology and teaching methods.
• Adaptation of the educational policy-making, planning and decision-making system with the new developments of the society in the field of cultural, political, and educational development.
• Future studies can investigate the technology literacy of language teachers and learners and the ways that might enhance teachers and learners’ understandings of these modern learning contexts.

## Data Availability Statement

The original contributions presented in the study are included in the article/supplementary material, further inquiries can be directed to the corresponding author.

## Ethics Statement

The studies involving human participants were reviewed and approved by the Xi’an University of Finance and Economics Academic Ethics Committee. The patients/participants provided their written informed consent to participate in this study.

## Author Contributions

The author confirms being the sole contributor of this work and has approved it for publication.

## Conflict of Interest

The author declares that the research was conducted in the absence of any commercial or financial relationships that could be construed as a potential conflict of interest.

## Publisher’s Note

All claims expressed in this article are solely those of the authors and do not necessarily represent those of their affiliated organizations, or those of the publisher, the editors and the reviewers. Any product that may be evaluated in this article, or claim that may be made by its manufacturer, is not guaranteed or endorsed by the publisher.
